# Modelling the impact of larviciding on the population dynamics and biting rates of *Simulium damnosum* (*s.l*.): implications for vector control as a complementary strategy for onchocerciasis elimination in Africa

**DOI:** 10.1186/s13071-018-2864-y

**Published:** 2018-05-29

**Authors:** Isobel Routledge, Martin Walker, Robert A. Cheke, Samir Bhatt, Pierre Baleguel Nkot, Graham A. Matthews, Didier Baleguel, Hans M. Dobson, Terry L. Wiles, Maria-Gloria Basañez

**Affiliations:** 1 MRC Centre for Outbreak Analysis and Modelling, Department of Infectious Disease Epidemiology, School of Public Health, Faculty of Medicine (St Mary’s campus), Imperial College London, Norfolk Place, London, W2 1PG UK; 2 London Centre for Neglected Tropical Disease Research (LCNTDR), Department of Pathobiology and Population Sciences, Royal Veterinary College, Hawkshead Lane, Hatfield, Hertfordshire, AL9 7TA UK; 3 London Centre for Neglected Tropical Disease Research (LCNTDR), Department of Infectious Disease Epidemiology, School of Public Health, Faculty of Medicine (St Mary’s campus), Imperial College London, Norfolk Place, London, W2 1PG UK; 4 Natural Resources Institute, Department of Agriculture, Health & Environment, University of Greenwich, Central Avenue, Chatham Maritime, Chatham, Kent, ME4 4TB UK; 5 Yaoundé Initiative Foundation, P.O. Box 3878, Messa, Yaoundé, Cameroon; 6 Yaoundé Initiative Foundation, Department of Life Sciences, Faculty of Natural Sciences (Silwood Park), Imperial College London, Ascot, Berkshire SL5 7PY UK

**Keywords:** Onchocerciasis, Vector control, Vector ecology, Mathematical modelling, Population dynamics, Alternative treatment strategy, Elimination, *Simulium damnosum* (*s.l*.), Africa

## Abstract

**Background:**

In 2012, the World Health Organization set goals for the elimination of onchocerciasis transmission by 2020 in selected African countries. Epidemiological data and mathematical modelling have indicated that elimination may not be achieved with annual ivermectin distribution in all endemic foci. Complementary and alternative treatment strategies (ATS), including vector control, will be necessary. Implementation of vector control will require that the ecology and population dynamics of *Simulium damnosum *(*sensu lato*) be carefully considered.

**Methods:**

We adapted our previous SIMuliid POPulation dynamics (SIMPOP) model to explore the impact of larvicidal insecticides on *S. damnosum* (*s.l*.) biting rates in different ecological contexts and to identify how frequently and for how long vector control should be continued to sustain substantive reductions in vector biting. SIMPOP was fitted to data from large-scale aerial larviciding trials in savannah sites (Ghana) and small-scale ground larviciding trials in forest areas (Cameroon). The model was validated against independent data from Burkina Faso/Côte d’Ivoire (savannah) and Bioko (forest). Scenario analysis explored the effects of ecological and programmatic factors such as pre-control daily biting rate (DBR) and larviciding scheme design on reductions and resurgences in biting rates.

**Results:**

The estimated efficacy of large-scale aerial larviciding in the savannah was greater than that of ground-based larviciding in the forest. Small changes in larvicidal efficacy can have large impacts on intervention success. At 93% larvicidal efficacy (a realistic value based on field trials), 10 consecutive weekly larvicidal treatments would reduce DBRs by 96% (e.g. from 400 to 16 bites/person/day). At 70% efficacy, and for 10 weekly applications, the DBR would decrease by 67% (e.g. from 400 to 132 bites/person/day). Larviciding is more likely to succeed in areas with lower water temperatures and where blackfly species have longer gonotrophic cycles.

**Conclusions:**

Focal vector control can reduce vector biting rates in settings where a high larvicidal efficacy can be achieved and an appropriate duration and frequency of larviciding can be ensured. Future work linking SIMPOP with onchocerciasis transmission models will permit evaluation of the impact of combined anti-vectorial and anti-parasitic interventions on accelerating elimination of the disease.

**Electronic supplementary material:**

The online version of this article (10.1186/s13071-018-2864-y) contains supplementary material, which is available to authorized users.

## Background

Human onchocerciasis, also known as river blindness, is a neglected tropical disease (NTD) [1, 2] caused by the filarial parasitic nematode, *Onchocerca volvulus.* The parasite spreads through the bite of blackflies within the genus *Simulium*, which act as disease vectors. Onchocerciasis has posed a serious public health and socio-economic burden for many countries in sub-Saharan Africa (SSA), and foci in Latin America and Yemen [3]. The disease continues to have devastating effects on the quality of life of those infected, over 99% of whom live in SSA [4].

Fortunately, great strides have been made in onchocerciasis control over the past 60 years. The Onchocerciasis Control Programme in West Africa (OCP, 1974–2002), initially an anti-vectorial intervention in 11 countries, averted 600,000 cases of preventable blindness and made 25 million hectares of land habitable and productive [5]. The African Programme for Onchocerciasis Control (APOC, 1995–2015), mainly an anti-parasitic intervention in the remaining endemic African countries, averted an estimated 17.4 million disability-adjusted life years (DALYs) [6]. Onchocerciasis control has been praised as one of the most cost-effective interventions of the last 100 years, both from the perspectives of public health [7] and economic development [8, 9]. Mass drug administration (MDA) through community-directed treatment with ivermectin (CDTI) has saved an estimated 500,000 DALYs every year at US$7 per DALY [6, 10]. None of these programmes, however, led to regional elimination of *O. volvulus* by the time of their closure.

Current World Health Organization (WHO) targets are to eliminate the disease in selected African countries by 2020, and in at least 80% of onchocerciasis endemic African countries by 2025 [11, 12]. As the focus shifts towards elimination, there is a need to consider whether the current control strategy (mainly annual CDTI) is satisfactory to achieve elimination in all epidemiological contexts. Evidence suggests that complementary and/or alternative treatment strategies (ATS) [13] should be considered for foci with high pre-control transmission intensity (hyper- and holoendemic areas with baseline microfilarial prevalence in excess of 60%) [14, 15], where sub-optimal responses to ivermectin have been documented [16, 17], and where co-endemicity with *Loa loa* (African eye-worm) - particularly in the case of hypoendemic onchocerciasis - contraindicates ivermectin MDA due to the risk of severe adverse events (SAEs) when treating individuals with very high *L. loa* microfilaraemia [18, 19].

Localized vector control through ground-based larviciding has been recommended as one of the ATS to be carried out in combination with CDTI, or with test-and-treat/not-treat strategies where co-endemicity with *L. loa* is a concern [13, 20]. However, many questions remain as to where, when, how frequently, for how long and how focal vector control should be implemented. For instance, Uganda is the only country in SSA whose onchocerciasis elimination programme currently combines vector control and ivermectin treatment [21], yet the Ugandan experience may not be directly applicable to other African settings given the very specific ecological requirements of the local *S. neavei* vector (whose immature stages have a phoretic association with freshwater crabs). However, *S. damnosum *(*sensu lato*) is also important in Uganda.

It is, therefore, essential to understand vector ecology and population dynamics when deciding where, when and how to implement larviciding programmes in focal areas. Members of the *S. damnosum* (*s.l*.) complex, which transmit *O. volvulus* in most of Africa, are sub-categorised into numerous cytospecies, which have differing vector competences, vectorial capacities and could have different susceptibilities to insecticides. Therefore, cytospecies identities are important to consider when designing vector control strategies. A separation on ecological and epidemiological grounds is often made between cytospecies which reside in savannah habitats, such as *S. damnosum *(*sensu stricto*) and *S. sirbanum,* and those in forested areas, such as *S. sanctipauli* and *S. yahense*.

However, onchocerciasis transmission dynamics and control models such as EPIONCHO and ONCHOSIM do not currently consider details of the population dynamics of the vectors, but tend to use only a simulated reduction in vector biting rates when modelling vector control, rather than modelling explicitly the effect on the vector population of killing the simuliid larvae [22]. This could lead to misleading results when considering the epidemiological impact of focal vector control strategies because *Simulium* population dynamics and vector ecology have important effects on the implementation and outcomes of several aspects of vector control including optimal timing (regarding vector and parasite), frequency and duration, in addition to potential nonlinear effects due to the density-dependent processes that regulate vector and parasite abundance.

In this paper we use our previously developed SIMuliid POPulation dynamics (SIMPOP) model [23] to investigate under which contexts focal vector control would be successful in reducing adult vector biting rates substantially and to identify how frequently and for how long vector control would need to be carried out to effect substantive reductions in blackfly vector biting.

## Methods

### Data sources

#### OCP savannah site: Asubende, Ghana

This dataset, from an onchocerciasis hyperendemic savannah region located in the eastern part of the southern extension of OCP on the Pru river in Ghana, comprises daily biting rates (DBRs, the number of bites per person per day) from human landing catches recorded between August 1987 and March 1988. Larviciding started in the area in January 1986 but was interrupted in June 1987 and resumed on 10 February 1988 due to ongoing trials of the impact of ivermectin MDA on transmission [24]. Here *S. damnosum* (*s.s*.) is the dominant vector but *S. sirbanum* is also found. This dataset was used to fit the model and estimate parameters pertaining to savannah settings as listed in Table 1.

**Table 1:** Parameters of the SIMPOP model that were estimated by approximate Bayesian computation (ABC)

Notation	Definition and units	Prior mean values and assumed distribution (parameters or range)	Posterior mean	[95% Credible Interval]	Reference
*T*	Air temperature (°C)	Asubende (Ghana): *T* ∼ *Normal* (mean = 29, SD = 3) Sanaga (Cameroon): *T* ∼ *Normal* (mean = 29, SD = 3)	Asubende: 27.9; Sanaga: 26.8	Asubende: [24.3–32.3]; Sanaga: [20.8–33.7]	[23]
*T*_*W*_	Water temperature (°C)	Asubende (Ghana): 27 (25–33) Sanaga (Cameroon): 25 (22–29) Bioko (Equatorial Guinea): 24 (23–25) *T*_*W*_ = 0.9844 *T* − 1.0352		Derived from estimated air temperature (above), Asubende: 26.4; Sanaga: 25.3	[59] [29] [23]
${\mu}_L^0$	Background per capita mortality rate of larvae (day^–1^)	${\mu}_L^0\sim Normal\kern0.5em \left(\mathrm{mean}=0.27,\kern0.5em \mathrm{SD}=0.05\right)$	Asubende: 0.24; Sanaga: 0.25	Asubende: [0.16–0.32]; Sanaga: [0.17–0.34]	[23]
*μ*_*P*_	Per capita mortality rate of pupae (day^–1^)	*μ*_*P*_ ∼ *Normal* (mean = 0.1, SD = 0.05)	Asubende: 0.1; Sanaga: 0.1	Asubende: [0.0–0.2]; Sanaga: [0.02–0.2]	[23]
${\overline{\mu}}_V$	Per capita loss rate of adult female flies (mortality + emigration) (day^–1^)	Asubende: a relationship between adult fly mortality and air temperature [23] was used, and the loss term (*Em*) was estimated: $$ {\displaystyle \begin{array}{l}{\overline{\mu}}_V={\mu}_V(T)+ Em\\ {}{\mu}_V(T)=0.0027{T}^2-0.163T+2.602\\ {} Em\sim Uniform\left(0,0.5\right)\end{array}} $$	Asubende: *Em* = 0.17 ${\overline{\mu}}_V=0.33$	[0.04–0.37] [0.2–0.53]	This study
		Sanaga: a single term is estimated ${\overline{\mu}}_V\sim Uniform\left(0,0.5\right)$	Sanaga: ${\overline{\mu}}_V=0.32$	[0.2–0.45]	This study
*ε*_*L*_	Efficacy of larviciding (1 minus the proportion of larvae surviving one day after deploying the insecticide)	*ε*_*L*_ ∼ *Normal* (mean = 0.8, SD = 0.2)	Asubende: 0.99; Sanaga: 0.96	Asubende: [0.979–0.997]; Sanaga: [0.92–0.99]	[26]
*g*	Length of gonotrophic cycle (days)	*g~Normal* (mean-3.5, SD=0.25)	Asubende: 3.27; Sanaga: 3.45	Asubende: [2.89–3.61]; Sanaga: [2.96–3.86]	[23]
$\left(\frac{H}{h}\right)$	Human population density/human blood index	$\left(\frac{H}{h}\sim Uniform\left(100,1000\right)\right)$	Asubende: 586; Sanaga: 535	Asubende: [252–933]; Sanaga: [190–935]	[46, 49]
DBR*	Pre-intervention equilibrium, daily biting rate (bites/person/day)	Asubende: *DBR*^∗^ ∼ *Normal*(mean = 220, SD = 30) Sanaga: *DBR*^∗^ ∼ *Normal*(mean = 352, SD = 64)	Asubende: 213; Sanaga: 333	Asubende: [117–270]; Sanaga: [256–435]	[26, 49]

#### OCP pilot studies at savannah sites: Burkina Faso/Côte d’Ivoire

This dataset [25] contains weekly averages of DBRs recorded at three savannah sites (Léraba Bridge, Chaussée Niakaramandougou and Naniénavogo) in Burkina Faso/Côte d’Ivoire during 1975 in the south-western section of the original OCP control area, where aerial larviciding was introduced. These data came from early pilot field trials where vector control had not previously been carried out and were measured by landing catches on human attractants. This dataset was used for model validation purposes regarding savannah settings.

#### Focal vector control in forested sites: Sanaga Valley, Cameroon

In 2005, the Yaoundé Initiative Foundation (YIF) began to pilot integrated vector control which targeted vectors of both malaria and onchocerciasis in the Sanaga Valley in Cameroon [26], a forest area where the principal vector of onchocerciasis is *S. squamosum* B [27]. Under this integrated vector control trial, larviciding occurred for 3 applications every 10 days at two sites upstream of the Kikot falls (Lenouck and Ntol), and was carried out in a focal manner, delivered by pirogue (small boat) [26]. Samples of vegetation were taken from the Kikot falls to identify the reduction in larval populations, and adult flies were caught using sticky traps (rather than human attractants), similar to those described in [28]. Since these data are not based on DBRs, a directly proportional relationship between fly density on sticky traps and DBR that had been determined by the YIF field team in their trial was used, with 50 flies/trap/day being approximately equivalent to 160 bites/person/day (i.e. DBR ≈ 3.2 × daily fly density on sticky traps). These data were used for model fitting regarding forest settings (Table 1). Data also exist from antivectorial intervention in the Sanaga river, where larviciding was carried out in response to fly numbers, measured by the catches on the sticky traps. When fly numbers exceeded 30 flies/trap/day, larviciding was resumed, when it would be applied roughly every seven days until numbers of flies caught decreased below this value. These data were not used for model fitting due to the variable interval between applications dictated by reaching the chosen target vector density.

#### Focal vector controlled forested sites: Bioko

This dataset, recording weekly averages of DBRs during 2001 at three different sites (Musola, Barleycorn and Sampaca), was collected as part of APOC pilot studies on the effectiveness and feasibility of localized vector control in the elimination of onchocerciasis from Bioko, an island off the coast of Cameroon and part of Equatorial Guinea. Detailed collection methods were described in [29]. Bioko is covered by large swathes of forest, and the local onchocerciasis vectors (the Bioko form of *S. yahense* [30]) have now been eliminated from the island [31] (in addition to ivermectin MDA being implemented). This dataset also includes unpublished records of water temperature and pH at sites during the time of sampling (R. A. Cheke, unpublished data). This dataset was used for model validation regarding forest settings.

### SIMuliid POPulation dynamics model (SIMPOP)

Our previously described *S. damnosum* population dynamics model [23] was modified to consider the effect of larviciding regimes on the dynamics of the immature stages and on adult DBRs of *S. damnosum* (*s.l*.) female flies. SIMPOP is a compartmental, population-based deterministic model described by a system of ordinary differential equations (ODEs) representing the changes in number of simuliid eggs, larvae, pupae and adult flies with time. The developmental rates of the immature stages are water-temperature dependent. The model was refined to include a time-dependent excess larval mortality term due to exposure to larviciding insecticide, a flexible “loss function” that captures both adult fly mortality and emigration, and additional larval instar stages replacing a single larval compartment to better model developmental times from larvae to pupae. (Under the assumption of a single larval compartment these would be exponentially distributed, with larvae becoming pupae very quickly; the addition of larval compartments permits a more realistic progression, with developmental times approaching a gamma distribution).

**Fig. 1:**
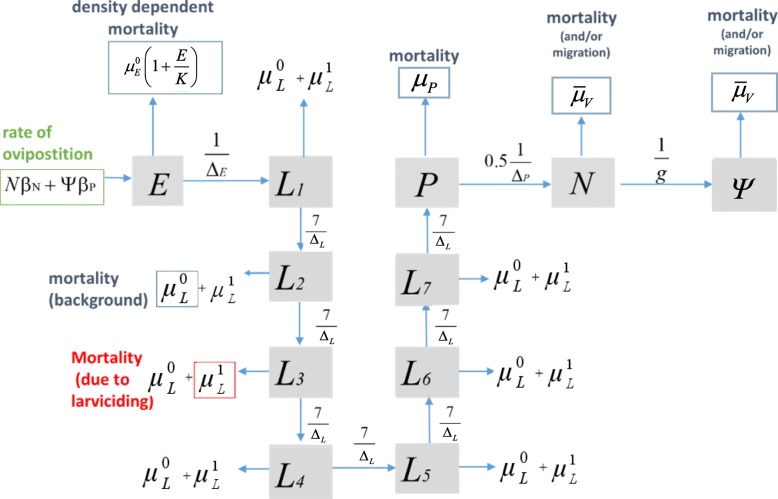
Flow diagram of the model for the population dynamics of *Simulium damnosum* (*s.l*.) (SIMPOP). Boxes represent life-cycle states (eggs, larval instar stages 1–7, pupae, nulliparous and parous adults), arrows represent movement in and out of those states

Figure 1 illustrates the flow diagram and equations (1) to (6) describe the SIMPOP model used in this study. State variables, mathematical expressions [other than equations (1)-(6)] and (fixed) model parameters are summarized in Table 2. Parameter values were either taken from [23] or, as stated above, estimated by fitting the model to data using approximate Bayesian computation (ABC) methods [32], and were informed from laboratory/field data in similar contexts (Table 1). We used the relationship between water temperature *T*_*W*_ and air temperature *T* given in [23], namely, *T*_*W *_= 0.9844 *T* - 1.0352. Following [23], the rates of change with respect to time of blackfly eggs (*E*), larvae (*L*), pupae (*P*) and adult (nulliparous, *N* and parous, *Ψ*) female flies are given by the following ODEs (indicating time, *t*, water temperature, *T*_*W*_ and air temperatue *T*, dependencies),

1 $$ \frac{dE\left(t,{T}_W\right)}{dt}=N\left(t,{T}_W,T\right){\beta}_N+\varPsi \left(t,{T}_W,T\right){\beta}_P-\frac{E\left(t,{T}_W\right)}{\Delta_E\left({T}_W\right)}-{\mu}_E^0\left(1+\frac{E\left(t,{T}_W\right)}{K}\right)E\left(t,{T}_W\right), $$

2 $$ {\displaystyle \begin{array}{c}\frac{dL_1\left(t,{T}_W\right)}{dt}=\frac{E\left(t,{T}_W\right)}{\Delta_E\left({T}_W\right)}-\left(\frac{7}{\Delta_L\left({T}_W\right)}+{\mu}_L^0+{\mu}_L^1\right){L}_1\left(t,{T}_W\right)\\ {}\frac{dL_i\left(t,{T}_W\right)}{dt}=\frac{7{L}_{i-1}\left(t,{T}_W\right)}{\Delta_L\left({T}_W\right)}-\left(\frac{7}{\Delta_L\left({T}_W\right)}+{\mu}_L^0+{\mu}_L^1\right){L}_i\left(t,{T}_W\right)2\le i\le 7\end{array}}, $$

3 $$ \frac{dP\left(t,{T}_W\right)}{dt}=\frac{7{L}_7\left(t,{T}_W\right)}{\Delta_L\left({T}_W\right)}-\frac{P\left(t,{T}_W\right)}{\Delta_P\left({T}_W\right)}-P\left(t,{T}_W\right){\mu}_P, $$

4 $$ \frac{dN\left(t,{T}_W,T\right)}{dt}=\frac{0.5P\left(t,{T}_W\right)}{\Delta_P\left(t,{T}_W\right)}-\left(\frac{1}{g(T)}+{\overline{\mu}}_V(T)\right)N\left(t,{T}_W,T\right), $$

5 $$ \frac{d\varPsi \left(t,{T}_W,T\right)}{d t}=\left(\frac{1}{g(T)}\right)N\left(t,{T}_W,T\right)-{\overline{\mu}}_V(T)\varPsi \left(t,{T}_W,T\right). $$

**Table 2:** State variables, expressions and (fixed) parameters of the SIMPOP model

Notation	Definition and units	Expression	Mean	Reference
*E*(*t*, *T*_*W*_)	Mean no. of simuliid eggs at time *t* and water temperature *T*_*W*_	Eqn. (1)	–	[23]
$$ {\displaystyle \begin{array}{l}{L}_1\left(t,{T}_W\right);{L}_i\left(t,{T}_W\right)\\ {}2\le i\le 7\end{array}} $$	Mean no. of 1st instar larvae and of 2nd to of 7th instar larvae at *t* and *T*_*W*_	Eqn. (2)	–	This study
*P*(*t*, *T*_*W*_)	Mean no. of pupae at *t* and *T*_*W*_	Eqn. (3)	–	[23]
*N*(*t*, *T*_*W*_, *T*)	Mean no. of nullipars at time *t*, water temperature *T*_*W*_ and air temperature *T*	Eqn. (4)	–	[23]
*Ψ*(*t*, *T*_*W*_, *T*)	Mean no. of parous flies at *t*, *T*_*W*_ and *T*	Eqn. (5)	–	[23]
*V*(*t*, *T*_*W*_, *T*)	Mean no. of vectors at *t*, *T*_*W*_ and *T*	*N*(*t*, *T*_*W*_, *T*) + *Ψ*(*t*, *T*_*W*_, *T*)	–	[23]
Δ_*E*_(*T*_*W*_)	Duration of egg stage at water temperature *T*_*W*_ (days)	11.493 exp(−0.0701*T*_*W*_)	–	[23]
Δ_*L*_(*T*_*W*_)	Duration of larval stage at *T*_*W*_ (days)	87.527 exp(−0.0785*T*_*W*_)	–	[23]
Δ_*P*_(*T*_*W*_)	Duration of pupal stage at *T*_*W*_ (days)	20.098 exp(−0.0699*T*_*W*_)	–	[23]
${\mu}_E^0$	Background per capita rate of eggs (day^–1^)	–	0.05	[23]
${\mu}_L^1$	Mortality rate of larvae due to larviciding (day-^1^)	$\left[-\ln \left(1-{\varepsilon}_L\right)-{\mu}_L^0\right]$	–	This study
*β*_*N*_	Per nulliparous fly rate of oviposition (day^–1^)	$\frac{\varepsilon_N\left\{\exp \left[-{\mu}_V(T)g(T)\right]\right\}}{g(T)}$	–	[23]
*β*_*P*_	Per parous fly rate of oviposition (day^-1^)	$\frac{\varepsilon_P{\mu}_V(T)}{\left\{\exp \left[{\mu}_V(T)g(T)\right]-1\right\}}$	–	[23]
*ε*_*N*_	Per capita mean no. of eggs per nulliparous fly	*S. damnosum* (*s.s*.)/*S.sirbanum* *S. squamosum*	432 492	[23, 62, 63]
*ε*_*P*_	Per capita mean no. of eggs per parous fly	*S. damnosum* (*s.s*.)/*S. sirbanum* *S. squamosum*	142 215	[23, 62, 63]
*DBR*	Daily biting rate (day^-1^)	Eqn. (6)	–	–

The equation for the daily biting rate, *DBR*, is,

6 $$ DBR\left(t,{T}_W,T\right)=\frac{V}{H}\left(\frac{h}{g(T)}\right)=\frac{N\left(t,{T}_W,T\right)+\varPsi \left(t,{T}_W,T\right)}{H}\left(\frac{h}{g(T)}\right), $$

where *V* is the total biting vector population, comprising nulliparous and parous female flies; *H* is the human population density, *h* is the proportion of blood meals taken on humans (or human blood index) and *g*(*T*) is the duration of the gonotrophic cycle (the average period between two consecutive blood meals) which would be influenced by environmental temperature; in practice we do not have an explicit expression for this relationship but we have included it in the equations above for the sake of completeness. We assume gonotrophic concordance, i.e. one blood meal for the development of one batch of eggs.

#### Addition of loss function

The adult mortality term ${\overline{\mu}}_V(T)$ represents an (estimated) composite parameter instead of a simple vector mortality rate, due to the possibility of adult fly migration in and out of the study sites, which has been observed in previous field studies in savannah settings [25, 33, 34]. For the modelling presented here we only considered emigration (parameter *Em*). In [23], a relationship between adult fly mortality and air temperature was established for Asubende, which was used here as part of the loss function for the savannah settings (Table 1). However, due to the context-specific nature of this parameter, this relationship was not used for forest adult fly mortality; instead the composite loss function was estimated for this setting.

#### Addition of larval instar stages

Seven larval instar stages were added, in place of a single larval compartment, better reflecting the life-cycle of blackfly aquatic stages [35]. This has been used to model increased realism in developmental times in other population dynamics models of insect disease vectors, for example *Anopheles gambiae* [36]. Otherwise a proportion of larvae leave the larval compartment much more quickly than occurs in reality; this is because the assumption of a constant rate of progression through model compartments leads to an exponential distribution of time spent in the larval compartment. This then affects the numbers that progress to the next compartment and the effect of larviciding on population dynamics.

#### Time-dependent mortality due to larviciding

An extra-mortality term for blackfly larvae was introduced, ${\mu}_L^1$, which represents excess larval mortality due to larviciding (in addition to the background larval mortality rate ${\mu}_L^0$). The model considers a larviciding regime carried out at regular intervals, with ${\mu}_L^1$ representing a near instantaneous death of blackfly larvae which come into contact with the larvicide. Larvicidal efficacy, *ε*_*L*_, is defined as 1 minus the proportion of larvae surviving one day after deploying the insecticide, reflecting the rapid activity of insecticides such as temephos, which has been shown to be highly efficacious against *S. damnosum* (*s.l*.) larvae at concentrations giving 0.05 mg/l at high river discharges (> 25 cubic metres/second) and at 0.1mg/l at low discharges (when insecticide carry is reduced) [37]. Although temephos efficacy has been shown to be variable in the field [37], laboratory studies on *S. damnosum* (*s.l*.) in Tanzania [38] have found an efficacy of 99–100%. Field studies of larvicidal efficacy during contemporary ground-based vector control strategies found values between 93–96% [26], whereas other models [39] have considered scenarios with 99% efficacy. In practice, inadequately implemented larviciding regimes or regimes in very difficult to reach areas are likely to have lower efficacies.

#### Re-estimation of carrying capacity, *K*

Following [23], and in order to stabilise the population, the density-dependent mortality of eggs is expressed in terms of the carrying capacity of adult vectors *K*, i.e. ${\mu}_E(K)={\mu}_E^0\left(1+\frac{E\left(t,T\right)}{K}\right)$, where ${\mu}_E^0$ is the background mortality rate of eggs. Due to the modifications of the model implemented here, a new relationship between carrying capacity, *K*, and the equilibrium number of adult (female) flies, V*, was derived by setting the equations for the blackfly population dynamics to zero to obtain equilibrium expressions for each stage (see equation 4.9 of [23]). Details of this derivation can be found in Additional file 1 (*Re-estimating carrying capacity, K*).

### Calibration of SIMPOP for different ecological/taxonomic contexts

Using the datasets described above, the model was calibrated for savannah and forest settings by fitting the model, respectively, to the Asubende and the Sanaga Valley data using Approximate Bayesian Computation (ABC) methods implemented with the *abc* R package [40]. The methods are fully described in Additional file 1 (*Description of approximate Bayesian computation for parameter estimation*).

Briefly, parameter sets were sampled from prior distributions informed by the published literature. Table 1 lists the estimated parameters and their prior distributions alongside the relevant references used to inform these priors. The majority of the parameters were given informative priors (i.e. those with values and ranges well established in the literature, e.g. larvicidal efficacy), whilst others (e.g. the adult fly loss function) were given uninformative/vague priors. Parameter sets drawn from these priors were inputted into SIMPOP to produce simulations of the mean number of bites per person per day over time and, from a Poisson distribution, to yield simulated datasets of the number of bites per person per day.

For each simulation, from a particular parameter set, summary statistics were computed from the simulated data and compared to summary statistics from the actual data using a distance measure defined by the Poisson log likelihood. Parameter sets yielding simulated datasets with log likelihoods sufficiently close (defined by an acceptance or threshold parameter, Additional file 1, *Description of approximate Bayesian computation for parameter estimation*) to the log likelihood of the observed data were considered as samples from the approximate posterior distribution (posterior). This was repeated for 1500 parameter sets drawn from the prior distributions. The version of ABC inference used for this analysis also employs a *post-hoc* machine-learning regression technique to improve the approximation of the posterior. In particular, we made use of neural networks [41] to correct for the imperfect match between the accepted and observed likelihoods. Rather than simply setting a tolerance threshold and rejecting a proportion of parameter values away from the observed likelihood, this approach considers how similar likelihoods of accepted parameter sets are to observed likelihoods. When just a tolerance is used, some parameters may be included which are quite far away from observed likelihood and useful information from the summary statistics is lost.

Parameter posteriors were summarised using the mean and the associated ninety five percent credible interval (95% CI). The model, calibrated either with data from Ghana (savannah setting) or Cameroon (forest setting) was then validated against the corresponding independent datasets Burkina Faso/Côte d’Ivoire (savannah) and Bioko (forest).

### Scenario analysis

Once SIMPOP was calibrated for *S. damnosum* (*s.s*.) savannah settings and *S. squamosum* B forest settings, the number of larvicide applications and the interval between larvicide applications were varied to evaluate effectiveness and identify optimal treatment strategies. Effectiveness was quantified by three outcome measures, namely, (i) the proportion of bites averted, defined as the proportion of bites prevented given the expected number of bites that would have been received during the treatment period in the absence of control; (ii) the proportional reduction in *DBR*, measured as 1 minus the ratio between the *DBR* one day after the cessation of larviciding and the endemic pre-intervention baseline *DBR*; (iii) the time to repopulation or bounce-back, defined as the number of days between one day after cessation of larvicidal operations and the vector biting rate returning to pre-intervention baseline levels. Hence, the most effective regimens were those with the highest proportion of bites averted, the greatest proportional reduction in *DBR*, and the longest time to bounce-back of adult fly populations.

### Exploring the effect of insecticide efficacy, endemic (pre-intervention) vector density and ecological factors on the impact of larviciding on adult fly biting rates

We explored the effect of varying individually several context-specific parameters on the output of the fitted model to determine their potential impact on ground-based larviciding control programmes in a wider range of contexts. These parameters were: (a) the pre-intervention baseline daily biting *rate*, DBR* (varied between 100 and 900 bites/person/day); (b) the air temperature (varied between 25 and 28 °C, and its corresponding variation in water temperature between 23.6 and 26.5 °C); (c) the length of the gonotrophic cycle (varied between 2.5 and 4 days); and (d) the larvicidal efficacy (varied between 50 and 99.5%). These parameters were chosen on the basis of either being important in the ecology and population dynamics of *Simulium* spp. (DBR*, air temperature and length of gonotrophic cycle), or being programmatically relevant (larvicidal efficacy; although intrinsic susceptibility to larvicidal insecticides may also be influenced by cytospecies-specific factors which were not considered explicitly here). Other parameters were also varied individually to explore their impact on model outputs (Additional file 1, *Sensitivity of model to changes in parameter values*). (Notice that we refer to observed daily biting rates as DBRs to denote the data, whereas we use *DBR* when referring to modelled biting rates; in particular, DBR* denotes the modelled pre-intervention baseline/equilibrium daily biting rate.)

## Results

### Calibration and validation

**Fig. 2:**
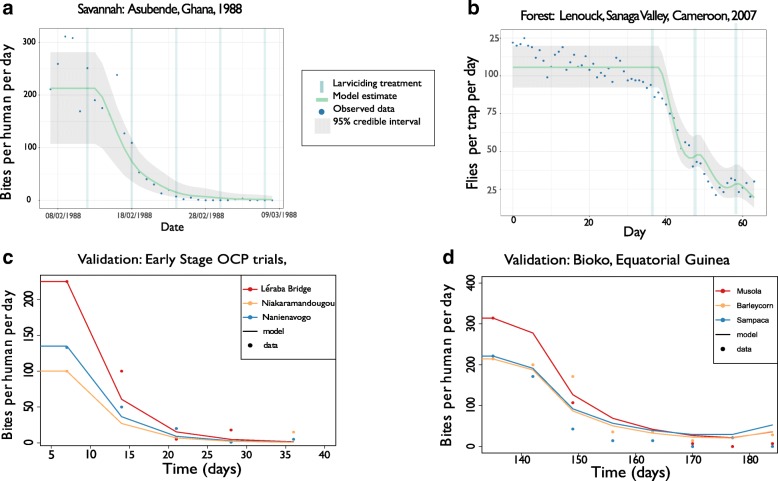
Model calibration and validation. **a** Calibration of model for savannah settings by fitting SIMPOP to data from Asubende, river Pru, Ghana, corresponding to 7-day intervals of aerial larviciding. **b** Calibration for forest settings by fitting the model to data from Lenouck, Sanaga river valley, Cameroon, corresponding to 10-day intervals of pirogue (boat)-based larviciding. **c** Validation of savannah-calibrated model against data from weekly (aerial) larviciding in OCP sites in Burkina Faso and Côte d’Ivoire. **d** Validation of forest-calibrated model against data from weekly larviciding by boat in forest areas of Bioko. In the validation datasets (**c, d**), local values of pre-intervention equilibrium *DBR* and air/water temperature were used but all remaining parameters were unchanged

The calibrated model captured the majority of observed DBRs within the 95% CIs for both savannah (dominant vector *S. damnosum* (*s.s*.)) and forest (dominant vector *S. squamosum* B) contexts (Fig. 2a, b). The validation, where only the pre-intervention equilibrium DBR*, air and water temperature were altered to reflect local conditions (Table 1), also captured well the observed data (Fig. 2c, d). Pairs plots (Additional file 1, *Pairs plots of parameter estimates*) suggested that none of the parameters were strongly correlated, with the exception of, for the forest parameterisation, the adult loss function $\left({\overline{\mu}}_V(T)\right)$ and the background (in the absence of larvicide) larval mortality rate $\left({\mu}_L^0\right)$, as the relationship between temperature and adult fly mortality is less well characterised for this context (i.e. compared to the savannah setting [23]), and as a result the two terms were not fully identifiable.

### Scenario analysis

#### Larvicidal efficacy and effectiveness of implementation scenarios

##### Efficacy and effectiveness in savannah settings

**Fig. 3:**
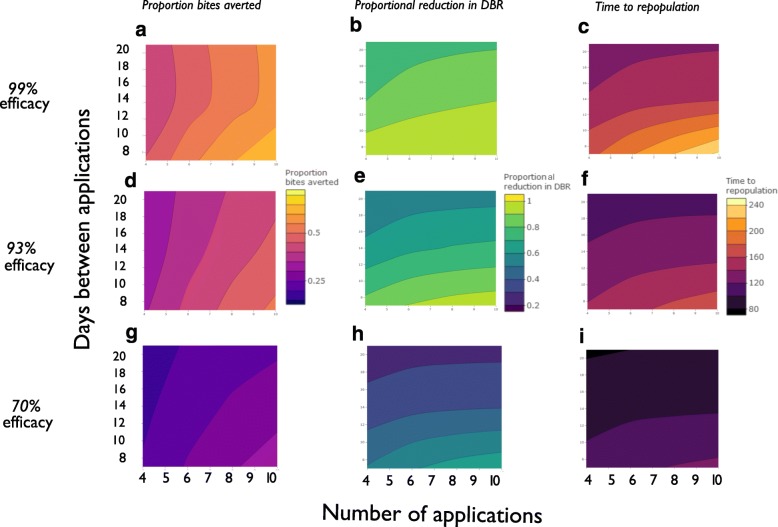
Scenario analysis (savannah settings). Impact of varying the number of larvicide applications (horizontal axes) and the interval between applications (in days, vertical axes) on three measures of effectiveness: (i) the proportion of bites averted during the intervention (vertical left-hand panels); (ii) the proportional reduction in *DBR* (vertical middle panels); (iii) the time taken to return to pre-intervention baseline *DBRs* (vertical right-hand panels). In **a**, **b** and **c** larvicidal efficacy is 99%. In **d**, **e** and **f** the results for 93% efficacy are presented. In **g**, **h** and **i** larvicidal efficacy is 70%. (For precise definitions of the effectiveness measurements see section on Scenario analysis in the main text. Equivalent results for the forest settings are presented in Additional file 1, *Results of scenario analysis*)

The mean of the estimated posterior of larvicidal efficacy in the Asubende study was 99% for the savannah-calibrated model. This model predicted a proportional reduction in *DBR* (one day after the last larvicidal treatment compared to one day before the first treatment) after 4 or 10 larviciding treatments every 7 days (i.e. weekly) of, respectively, 97% and 100%. Extending the interval between treatments to 14 days (i.e. every 2 weeks) and 21 days (i.e. every 3 weeks) resulted in lower *DBR* reductions, of 89% and 78%, respectively for 10 larvicidal applications (Fig. 3a).

Similarly, extending the interval between treatments from 7 to 14 days resulted in a smaller proportion of bites averted (60% compared to 69% following 10 treatments), and a faster rate of adult blackfly repopulation (158 compared to 244 days to repopulation following 10 treatments, indicative of a smaller reduction in *DBR*). Extending the between-application interval from 14 to 21 days resulted in little discernable difference in these effectiveness measures (Fig. 3a, b, c), with a similar proportion of bites averted and a moderately longer time to re-population (60% bites averted and 158 days to re-population for 10 treatments with 14-day intervals compared to 61% bites averted and 138 days to re-population for 10 treatments with 21-day intervals).

##### Efficacy and effectiveness in forest settings

The mean of the estimated posterior of larvicidal efficacy was 96% in the Sanaga Valley trial [26] for the forest-calibrated model. Predicted patterns of effectiveness for different larviciding implementation scenarios were similar to those for the savannah context and are presented in Additional file 1 (*Results of scenario analysis*).

##### Impact of larvicidal efficacy

Setting larvicidal efficacy to 93%, a more conservative but realistic value for local vector control during the pilot phase of implementation [26], the model predicted proportional reduction in *DBR* was 96% following 10 weekly treatments for both savannah (Fig. 3d, e, f) and forest contexts (Additional file 1, *Results of scenario analysis*). At a pre-intervention baseline *DBR* of 400 bites/person/day (the average equivalent DBR observed in three forest sites in South-West Cameroon [42] where focal vector control is being implemented by the CouNTDown consortium (http://www.countdownonntds.org/our-research/integrated-control-strategy-1-macro-vector/, L. Hamill, pers. comm.), this equates to a predicted reduction in *DBR* to approximately 16 bites/person/day. (It must be noted that although Matthews et al. [26] found efficacies ranging between 93 and 97%, we chose the more conservative option of 93% for a realistic implementation efficacy.)

At 70% larvicidal efficacy, representing greater programmatic difficulties, poorer implementation, implementation in more inaccessible areas, or indeed where efficacy is dwindling due to evolving insecticidal resistance, the measures of effectiveness were lower (Fig. 3g, h, i). With this efficacy, the model predicted that after a total of 10 larvicide applications at intervals of 7 days in a savannah setting, the *DBR* would be reduced by 67%, 33% of bites would be averted and the time for the adult blackfly population to re-populate to pre-intervention levels would be 125 days. The corresponding estimates in the forest context were 65%, 33% and 123 days, respectively (Additional file 1, *Results of scenario analysis*).

The results of the scenario analysis in relation to optimal and minimal intervention design requirements to achieve specified target results are summarised in Table 3. For a larvicidal efficacy of 93%, the minimum number of weekly treatments required to achieve at least a 95% reduction in DBR would be, respectively, 8 and 9 for savannah and forest contexts. For the same larvicidal efficacy, and to delay the time to bounce-back/repopulation (for the fly density to reach baseline levels) to at least 200 days, 16 and 8 weekly treatments would be required in savannah and forest contexts, respectively.

**Table 3:** Optimal (and minimum) intervention characteristics required to achieve target results

Intervention characteristics	Optimal		Minimum to reduce DBR by 95%		Minimum to delay repopulation or bounce back (to baseline levels) to 200 days after last treatment	
	Savannah	Forest	Savannah	Forest	Savannah	Forest
Larvicidal efficacy (number and frequency of applications)	100%	100%	92% (10 weekly treatments)	92% (10 weekly treatments)	96% (10 weekly treatments)	90% (10 weekly treatments)
Number of treatments (larvicidal efficacy, frequency)	10 (highest considered)	10 (highest considered)	8 (93% efficacy, weekly treatments)	9 (93% efficacy, weekly treatments)	16 (93% efficacy, weekly treatments)	8 (93% efficacy, weekly treatments)
Treatment frequency (larvicidal efficacy, number of applications)	7 days (shortest considered)	7 days (shortest considered)	7 days (93% efficacy, 10 treatments)	7 days (93% efficacy, 10 treatments)	4 days (93% efficacy, 10 treatments)	9 days (93% efficacy, 10 treatments)

### Pre-intervention daily biting rate, temperature and gonotrophic cycle length

**Fig. 4:**
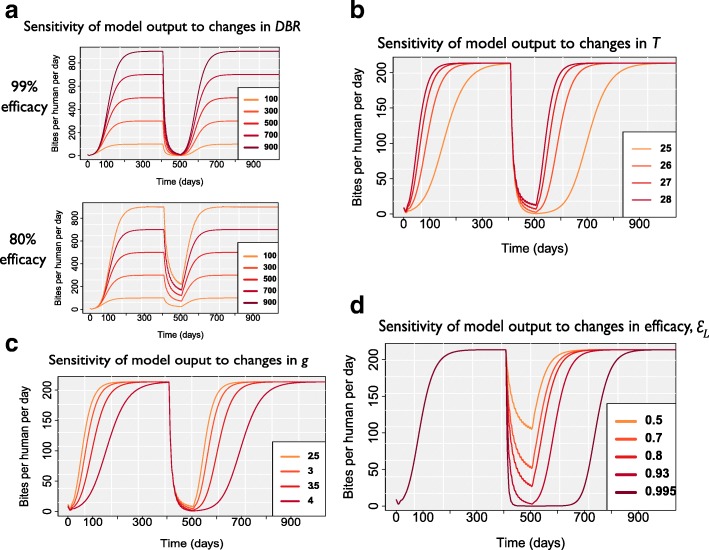
Sensitivity of model output to **a** pre-intervention daily biting rate DBR*; **b** air temperature, *T* (to which water temperature is related via *T*_*W*_=0.9844 *T*-1.0352, see [23]); **c** gonotrophic cycle length, *g*; and **d** larvicidal efficacy, *ε*_*L*_

When larvicidal efficacy is very high (99%), similar and substantial *DBR* reductions can be achieved regardless of initial pre-intervention biting rates (Fig. 4a, upper panel). However, when efficacy is reduced (to 80%), higher DBRs do not decline by as much (Fig. 4a, lower panel). Water temperature (related to air temperature) and gonotrophic cycle length also have important, non-linear effects on the effectiveness of larviciding on DBRs (Fig. 4b, c), with lower temperatures and therefore longer gonotrophic cycles resulting in slower rates of adult fly re-population. Figure 4d illustrates the impact of larvicidal efficacy on modelled *DBR.*

## Discussion

Vector biting rate is an important determinant of transmission intensity, endemicity level (measured in terms of *O. volvulus* microfilarial prevalence and intensity) and feasibility of elimination of onchocerciasis with the current CDTI strategy. In areas of high pre-intervention baseline infection endemicity, it has been suggested that vector control could be used as an adjuvant and complementary intervention strategy [13]. Modelling results using EPIONCHO and ONCHOSIM [43] suggest that in such settings, vector control could be used in conjunction with ivermectin MDA to enhance effectiveness or to consolidate the gains made towards the path of onchocerciasis elimination. However, in both models, vector control has been implemented somewhat crudely, by instantaneously reducing adult biting rates to given lower levels for a (usually prolonged) period. In practice, the application of larvicidal insecticides will effect dynamic changes in both aquatic and aerial blackfly stages, with adult fly populations recovering gradually after cessation of operation. Also, programmes considering the implementation of (local, ground-based) vector control may do so for variable periods depending, among other things, on seasonal feasibility of implementation and vector ecology. (For instance, ground-based larviciding targeted at *S. neavei* in the Kashoya-Kitomi focus of western Uganda was deployed monthly for 3 years for a total of 36 applications [21]. In the Bahr El Ghazal region of south western Sudan, temephos was applied for one annual transmission period, with ensuing reductions of 70% in vector biting and of 80% in transmission at sites with the highest pre-control levels [37]).

In order to investigate more realistic options for modelling vector control, we have extended our previous blackfly population dynamics model [23] to explore the effects of larviciding on the population dynamics and biting rates of *S. damnosum* (*s.l*.) in West/Central African savannah and forest contexts. The results suggest that when larvicidal efficacy is high, the blackfly DBRs could be reduced to very low levels after 10 weeks of larviciding, even in areas of high pre-intervention blackfly population density (indicative of conditions with a high propensity for onchocerciasis transmission). However, our model also indicates that the magnitude of DBR reduction would be very sensitive to the larvicidal efficacy that can be achieved. We explored a range of efficacy values because, in practice, final net efficacy will depend on the type of insecticide, the local ecological and accessibility conditions of the breeding sites, and the susceptibility of the aquatic stages of the local vector species (which could decrease under insecticide selection pressure). Locally-specific parameters such as water temperature did not affect the rate of DBR decline following larviciding at high larvicidal efficacies but did affect rates of adult blackfly repopulation. Hence, the effectiveness of short-term focal vector control programmes in sustaining low blackfly population biting densities is likely to vary with the local ecology and possibly also on the dominant simuliid cytospecies. Vector control is likely to be most effective when a high larvicidal efficacy can be ensured, where breeding site water temperatures are lower and life-cycles (including gonotrophic cycles) are longer (e.g. in more shaded breeding sites, those located at higher altitudes).

Even in the absence of concomitant treatment of the onchocerciasis-affected human population, vector biting rate plays an important role in the persistence of onchocerciasis. For a given simuliid vector competence, vectorial capacity (including the propensity to feed on humans) and the values of other vector- and parasite-dependent population biology parameters, there is a threshold biting rate (*TBR*) below which onchocerciasis would not persist endemically. This *TBR* is related to the basic reproduction number of the parasite in a given environment (*R*_0_), and for savannah *O. volvulus-S. damnosum* combinations, it has been estimated to vary between 230 and 2300 bites/person/year, with a mean of approximately 700–800 [44–47]. This would represent an average of 2 bites/person/day (and a range between 1 and 6) in the absence of anti-parasitic interventions, and could be even lower in forest *O. volvulus-S. damnosum* combinations due to higher vector competence [48] and stronger anthropophagy [49]. More likely, vector control will be deployed as a complementary treatment strategy, and modelling studies have indicated that in the presence of MDA treatment, the value of the *TBR* will increase, emphasizing the beneficial role of vector control. These predicted shifts towards higher *TBR* values highlight avenues for accelerating elimination of the infection by including vector control, even when conducted at a relatively low level of effectiveness [47]. In the following sections, we discuss the role of larvicidal efficacy, vector ecology and overall vector control effectiveness.

### Larvicidal efficacy

The field efficacy of a larviciding approach is crucial to delivering effective vector control. Even larvicides such as temephos with near 100% efficacy in the laboratory [38] will inevitably have somewhat lower operational efficacy. Recent pilot focal vector control schemes have achieved efficacies of 93–96% [26] and for such high operational efficacies, our modelling results indicate that weekly larviciding for 10 weeks would reduce DBRs to very low levels regardless of the pre-control population density of blackflies. However, at lower larvicidal efficacies, and specifically in settings with high blackfly population densities, reductions in DBRs may never reach epidemiologically acceptable levels (particularly with respect to reducing nuisance biting to tolerable levels or increasing the *TBR* sufficiently to increase the likelihood of elimination) even after longer periods of vector control. It is reasonable to assume that high reported operational efficacies could be replicated elsewhere with careful planning (including consideration of the seasonality of breeding sites, river volume and flow, and the spatial spread of insecticide) and judicious implementation (including monitoring of insecticidal efficacy). Nonetheless, achieving and sustaining high operational/field efficacy is a priority when designing larviciding schemes. Therefore, measures for monitoring efficacy and responding to emerging larvicidal resistance should be put in place from the outset. Resistance to temephos and other larvicides arose rapidly during the aerially-delivered larvicidal applications during the OCP [50–52], highlighting the importance of careful monitoring as well as of forward planning and implementation of larvicide rotation schedules should the need arise. Efficacy can be monitored through sampling and measuring density of vector larvae at baseline before intervention in control and treated areas and 24 hours, 48 hours and then weekly for several months following treatment, again in control and treatment areas [53]. However, unlike mosquito larval monitoring, density for simuliids is generally measured on a 5-point abundance scale, from - to ++++, referring to absent, scarce, few, common, heavy, due to the difficulties in counting individual simuliid larvae [54].

### Ecology and effectiveness

The time taken for DBRs to return to pre-intervention levels following cessation of larviciding varies notably with adult and larval mortality and air/water temperature (see Additional file 1, *Sensitivity of model to changes in parameter values*)*,* indicating that the effectiveness in sustaining reductions in blackfly population densities will vary among locales. The forest parameterisation of the model resulted in greater proportional reductions in the *DBR* following larviciding, despite a lower estimated efficacy. In addition, the effects of larviciding were sustained for longer than in the model calibrated with savannah simuliid species, with longer times to repopulation (again, despite the lower estimated larvicidal efficacy). This is likely to be the result of lower temperatures in forested areas affecting life-cycle and gonotrophic cycle length as our estimates did not reveal large differences in adult or larval mortality. These results are promising for controlling vectors in (forest) loiasis-onchocerciasis co-endemic areas where CDTI is not effective or cannot be implemented because of the risk of SAEs following ivermectin treatment of individuals with high *L. loa* microfilaraemia [18, 19]. However, implementation of larviciding may be more difficult in forest compared to savannah areas because the potentially more difficult access to rivers and tributaries. Hence, in such areas it may be harder to achieve high levels of field/operational efficacy. Given that our model highlights the importance of larvicidal efficacy on achieving substantial *DBR* reductions, this is a very important consideration, and one which requires expertise from field practitioners and vector ecologists. Additionally, further research needs to be conducted linking SIMPOP and EPIONCHO [22] to explore the minimum level of larvicidal efficacy that should be aimed at when deploying intervention packages including both human treatment and vector breeding site treatment.

### Future directions

We are planning to integrate SIMPOP with EPIONCHO, to permit detailed exploration of the impact of vector larviciding on onchocerciasis transmission dynamics, control and elimination when chemotherapeutic interventions are combined with vector control. Modelling projections based on distribution of ivermectin [43] indicate that onchocerciasis elimination will not be achieved in reasonable timescales in hyperendemic/holoendemic areas, where blackfly biting is very intense and, therefore, vector control has been proposed as an ATS to accelerate progress towards elimination (and reduce nuisance biting) [13]. As discussed, the impact of vector control has generally been modelled (but see [55]) by reducing the annual biting rate of the blackfly vector population over the duration of control. This highly simplified approach does not lend itself to modelling explicitly the effect of larviciding (at different durations and frequencies) on blackfly population dynamics. Hence, the model presented here could contribute to improving projections of the impact of suites of interventions targeting onchocerciasis elimination. These suites could include, among others, optimisation of the frequency and timing of microfilaricidal treatment (e.g. MDA with ivermectin or moxidectin) in relation to natural vector seasonality [56] and vector control; the combination of available and novel macrofilaricides (e.g. anti-*Wolbachia* therapies) with vector control (as currently being trialled by the CouNTDown Consortium), and the addition of vector control in areas with suboptimal responses to ivermectin [57].

The model presented here for forest areas does not distinguish between adult mortality or net egress/ingress to and from neighbouring sites. However, assuming that the relationship between temperature and adult fly mortality developed for the Asubende savannah context [23] holds generally, including for forest contexts, the mean excess loss due to reasons other than mortality (i.e. emigration) could be estimated. Reinvasion of vectors from surrounding areas not under vector control was commonplace in OCP savannah sites [33, 34], justifying its incorporation into the model presented here. Nonetheless, designs of control areas should consider risk of migration/source-sink population dynamics. Future models, if spatially structured, could potentially model the movement of vectors among proximate populations and transmission zones and evaluate the impact this would have on vector control.

Field studies at key sites would provide important information for planning vector control. Data on the distribution of *S. damnosum* (*s.l*.) cytotaxa across Africa is somewhat limited, with the exception of some detailed country-specific studies [58–60]. This is important as different cytotaxa have varying vectorial capacities, fecundities, mortalities and habitats. Some may be of much greater concern for onchocerciasis transmission. Given the findings of different repopulation rates in different contexts, these field data would be important to capture locale-specific population dynamics, permitting model calibration for a variety of ecological settings.

## Conclusions

Our modelling findings indicate that focal vector control is likely to be effective in breeding sites where a high larvicidal efficacy can be achieved and maintained, applications can be delivered regularly and ideally weekly (in *S. damnosum* (*s.l*.) breeding sites), and sustained durations can be ensured. In addition to these programmatic factors, ecological features such as lower breeding site water temperatures, longer blackfly life-cycles, or longer gonotrophic cycles in the dominant *Simulium* cytospecies may also increase effectiveness. The refined SIMPOP model developed here has clear applications to the design of blackfly control strategies in African forest and savannah settings. Future work to integrate the model into *O. volvulus* transmission dynamics models, particularly EPIONCHO, will permit evaluation of the epidemiological impact of combined anti-vectorial and anti-parasitic interventions aiming at controlling and eliminating human onchocerciasis.

## Additional file

Additional file 1: Description of the calculation of carrying capacity, approximate Bayesian computation (ABC) methods and sensitivity/scenario analyses. Re-estimating carrying capacity, *K*. Description of approximate Bayesian computation for parameter estimation. Sensitivity of model to changes in parameter values. Pairs plots of parameter estimates. Results of scenario analysis. (PDF 641 kb)

## Abbreviations

95% CI: Ninety five percent credible interval
ABC: Approximate Bayesian computation
APOC: African Programme for Onchocerciasis Control
ATS: Alternative treatment strategy
CDTI: Community-directed treatment with ivermectin
DALY: Disability-adjusted life year
DBR: Daily biting rate
MDA: Mass drug administration
NTD: Neglected tropical disease
OCP: Onchocerciasis Control Programme in West Africa
ODE: Ordinary differential equation
*R*_0_: Basic reproduction ratio
*s.l*.: *sensu lato*
*s.s*.: *sensu stricto*
SAE: Severe adverse event
SIMPOP: SIMuliid POPulation dynamics model
SSA: Sub-Saharan Africa
TBR: Threshold biting rate
US$: United States dollar
WHO: World Health Organization
YIF: Yaoundé Initiative Foundation
