# Supplementary material for: Modelling the impact of larviciding on the population dynamics and biting rates of Simulium damnosum (s.l.): implications for vector control as a complementary strategy for onchocerciasis elimination in Africa
Source: Parasit Vectors. 2018 May 29;11:316. doi: 10.1186/s13071-018-2864-y (PMC5972405; doi:10.1186/s13071-018-2864-y)
Supplement: Supplementary file 1 — Description of the calculation of carrying capacity, approximate Bayesian computation (ABC) methods and sensitivity/scenario analyses. Re-estimating carrying capacity, K. Description of approximate Bayesian computation for parameter estimation. Sensitivity of model to changes in parameter values. Pairs plots of parameter estimates. Results of scenario analysis. (PDF 641 kb) [file 13071_2018_2864_MOESM1_ESM.pdf]

Modelling the impact of larviciding on the population dynamics and biting rates of *Simulium damnosum* s.l.: implications for vector control as a complementary strategy for onchocerciasis elimination in Africa

Isobel Routledge[1], Martin Walker[2,3], Robert A. Cheke[3,4], Samir Bhatt[1], Pierre Baleguel Nkot[5], Graham A. Matthews[6], Didier Baleguel[5], Hans M. Dobson[4], Terry L. Wiles[5,6], Maria-Gloria Basañez[1,3,*]

1 MRC Centre for Outbreak Analysis and Modelling, Faculty of Medicine, Department of Infectious Disease Epidemiology, Imperial College London, London, UK

2 London Centre for Neglected Tropical Disease Research, Department of Pathobiology and Population Sciences, Royal Veterinary College, Hatfield, UK

3 London Centre for Neglected Tropical Disease Research, Faculty of Medicine, Department of Infectious Disease Epidemiology, Imperial College London, London, UK

4 Natural Resources Institute, Department of Agriculture, Health & Environment, University of Greenwich, Chatham, UK

5 Yaoundé Initiative Foundation, Yaoundé, Cameroon

6 Yaoundé Initiative Foundation, Faculty of Natural Sciences, Department of Life Sciences (Silwood Park), Imperial College London, London UK

[*]Corresponding author: Maria-Gloria Basáñez (m.basanez@imperial.ac.uk)

Additional file 1

Description of the calculation of carrying capacity, approximate Bayesian computation (ABC) methods and sensitivity/scenario analysis

Contents

1. Re-estimating carrying capacity, *K*
2. Description of approximate Bayesian computation for parameter estimation
3. Sensitivity of model to changes in parameter values
4. Pairs plots of parameter values
5. Results of scenario analysis
6. Supplementary references

1. Re-estimating carrying capacity, *K*

Due to the extension of the original model described in Cheke et al. [1] to include additional larval instar stages, an expression for the carrying capacity was re-derived by solving the model presented in the equations (1) to (5) of the main text at equilibrium, i.e., $\frac{dE}{dt}=\frac{dL_i}{dt}=\frac{dP}{dt}=\frac{dN}{dt}=\frac{d\Psi}{dt}=0$, to obtain steady state solutions $(E^*, L_i^*, P^*, N^*, \Psi^*)$. In the following we omit the time and temperature dependencies to simplify notation.

$$\frac{d\Psi}{dt}=0 \Rightarrow N^* = g\,\bar{\mu}_V \Psi^*$$
$$\frac{dN}{dt}=0 \Rightarrow P^* = 2\Delta_P\left(1+g\,\bar{\mu}_V\right)\bar{\mu}_V \Psi^*$$
$$\frac{dP}{dt}=0 \Rightarrow L_7{}^* = \left(\frac{\Delta_L}{7}\right)\left(\frac{1}{\Delta_P}+\mu_P\right)P^*$$

Substituting P^*,

$$\frac{dP}{dt}=0 \Rightarrow L_7{}^* = 2\left(\frac{\Delta_L}{7}\right)\left(1+\Delta_P\mu_P\right)\left(1+g\,\bar{\mu}_V\right)\bar{\mu}_V\Psi^*$$
$$\frac{dL_{i+1}}{dt}=0 \Rightarrow L_i^* = \left[1+\left(\frac{\Delta_L}{7}\right)\left(\mu_L^0+\mu_L^1\right)\right]L_{i+1}{}^* \quad 1 \le i \le 6$$
$$\frac{dL_1}{dt}=0 \Rightarrow E^* = 2\Delta_E\left[1+\left(\frac{\Delta_L}{7}\right)\left(\mu_L^0+\mu_L^1\right)\right]^7\left(1+\Delta_P\mu_P\right)\left(1+g\bar{\mu}_V\right)\bar{\mu}_V\Psi^*$$

$$\text{Let } \omega = 2\Delta_E\left[1+\left(\frac{\Delta_L}{7}\right)\left(\mu_L^0+\mu_L^1\right)\right]^7\left(1+\Delta_P\mu_P\right)\left(1+g\bar{\mu}_V\right)\bar{\mu}_V$$

$$\frac{dE}{dt}=0 \Rightarrow N^*\beta_N + \Psi^*\beta_P = \frac{E^*}{\Delta_E}+\mu_E^0\left(1+\frac{E^*}{K}\right)E^*$$
$$E^* = \omega\Psi^*$$
$$N^* = g\bar{\mu}_V\Psi^*$$

Re-arranging to obtain expressions for the equilibrium number of parous flies, nullipars and total adult female flies, and the expression for carrying capacity as a function of the equilibrium number of flies,

$$\Psi^* = \frac{K\left[\beta_N g\bar{\mu}_V + \beta_P - \left(\frac{\omega}{\Delta_E}\right) - \mu_E^0\omega\right]}{\mu_E^0\omega^2}$$

$$N^* = \frac{Kg\bar{\mu}_V\left[\beta_N g\bar{\mu}_V + \beta_P - \left(\frac{\omega}{\Delta_E}\right) - \mu_E^0\omega\right]}{\mu_E^0\omega^2}$$

$$V^* = N^* + \Psi^* = \frac{K\left(1 + g\bar{\mu}_V\right)\left[\beta_N g\bar{\mu}_V + \beta_P - \left(\frac{\omega}{\Delta_E}\right) - \mu_E^0\omega\right]}{\mu_E^0\omega^2}$$

$$K = \frac{V^*\mu_E^0\omega^2}{\left(1 + g\bar{\mu}_V\right)\left[\beta_N g\bar{\mu}_V + \beta_P - \left(\frac{\omega}{\Delta_E}\right) - \mu_E^0\omega\right]}$$

For a given baseline egg mortality μ_E^0 we can calculate the egg mortality at equilibrium density $\mu_E(E^*)$ (i.e. after the excess mortality due to density dependence has taken place) as follows,

$$\mu_E(E^*) = \mu_E^0\left(1 + \frac{E^*}{K}\right) = \mu_E^0\left(1 + \frac{\omega\Psi^*}{K}\right) = \mu_E^0\left[1 + \frac{\beta_N g\bar{\mu}_V + \beta_P - \left(\frac{\omega}{\Delta_E}\right) - \omega\mu_E^0}{\omega\mu_E^0}\right] =$$

$$= \left(\frac{\beta_N g\bar{\mu}_V}{\omega}\right) + \left(\frac{\beta_P}{\omega}\right) - \left(\frac{1}{\Delta_E}\right)$$

Note that this expression is independent of μ_E^0, and therefore, density-dependent egg mortality will stabilize the blackfly population regardless of the value of the baseline egg mortality rate.

2. Description of approximate Bayesian computation for parameter estimation

Parameter estimation was undertaken using an approximate Bayesian computation (ABC) technique implemented using the abc package in the R programming environment. A detailed description of ABC methods is given in the published package description [2] and summarised here for clarity. Approximate Bayesian computation techniques approximate the posterior probability distribution of a set of parameters, here referred to generically as θ. A set of parameter values θ_i is sampled from its prior distribution to simulate a new data set D_i. This is repeated n times to generate D_1, D_2 ,…, D_n datasets. A single summary statistic or $S(D_i)$ (or a set of summary statistics) is computed from each simulated dataset and compared to the summary statistics obtained from the actual data $S(D_0)$ using a Euclidean distance measure, d. Here our summary statistic (calculated for the real and simulated datasets) was the Poisson negative likelihood of either the observed or simulated data. The goal of ABC is to select only parameter sets that yield distance measures between observed and simulated datasets that are below a threshold (i.e. the simulated data are suitably close to the observed data). This threshold is defined by a tolerance rate which determines the percentage of samples which are accepted. We used a tolerance of 0.3 from 1,500 simulations of 8 parameters drawn from independent prior distributions. The accepted simulations are considered a sample from the approximate posterior distribution.

The version of ABC inference used for this analysis also employs a *post hoc* machine learning regression technique to improve the approximation of the posterior. In particular, we made use of neural networks [3] to correct for the imperfect match between the accepted, $S(D_i)$, and observed summary statistics (likelihoods), $S(D_0)$, using the following non-linear regression equation,

$$\theta_i = m\big(S(D_i)\big) + \epsilon_i$$

Here m represents a non-linear regression function based on the nnet package [4], which fits a specified number of neural networks (we used the default 10 networks) and takes the median value and ϵ_i, a random error with variance σ. Parameter sets that closely match the target summary statistics (i.e. have a small random error term) are given more weight. A weighted sample from the posterior distribution is then obtained from the θ_is, having been corrected using the estimate $\hat{m}(\cdot)$ and $\hat{\epsilon}_i$ [5]. We also applied a correction for heteroscedasticity,

$$\theta_i^* = \hat{m}(S(D_0)) + \frac{\hat{\sigma}(S(D_0))}{\hat{\sigma}(S(D_i))}\hat{\epsilon}_i$$

where $\hat{\sigma}(\cdot)$ is the estimated conditional standard deviation [3]. Using a single summary statistic (as employed here), $\hat{\sigma}(S(D_0)) = 0$ and therefore $\theta_i^* = \hat{m}(S(D_0))$.

3. Sensitivity of model to changes in parameter values

Table S1 Parameters varied in sensitivity analysis

Parameter	Values considered				
$\bar{\mu}_V$	0.05	0.1	0.15	0.2	0.25
ε_L	0.5	0.7	0.8	0.93	0.995
μ_P	0	0.05	0.1	0.15	–
μ_L^0	0.225	0.25	0.275	0.3	–
g	2.5	3	3.5	4	–
T	25	26	27	28	–
T_W [a]	23.6	24.6	25.5	26.5	–
DBR	100	300	500	700	900
$\left(\frac{H}{h}\right)$	50	100	200	400	800

[a] $T_W = 0.9844\,T - 1.0352$.

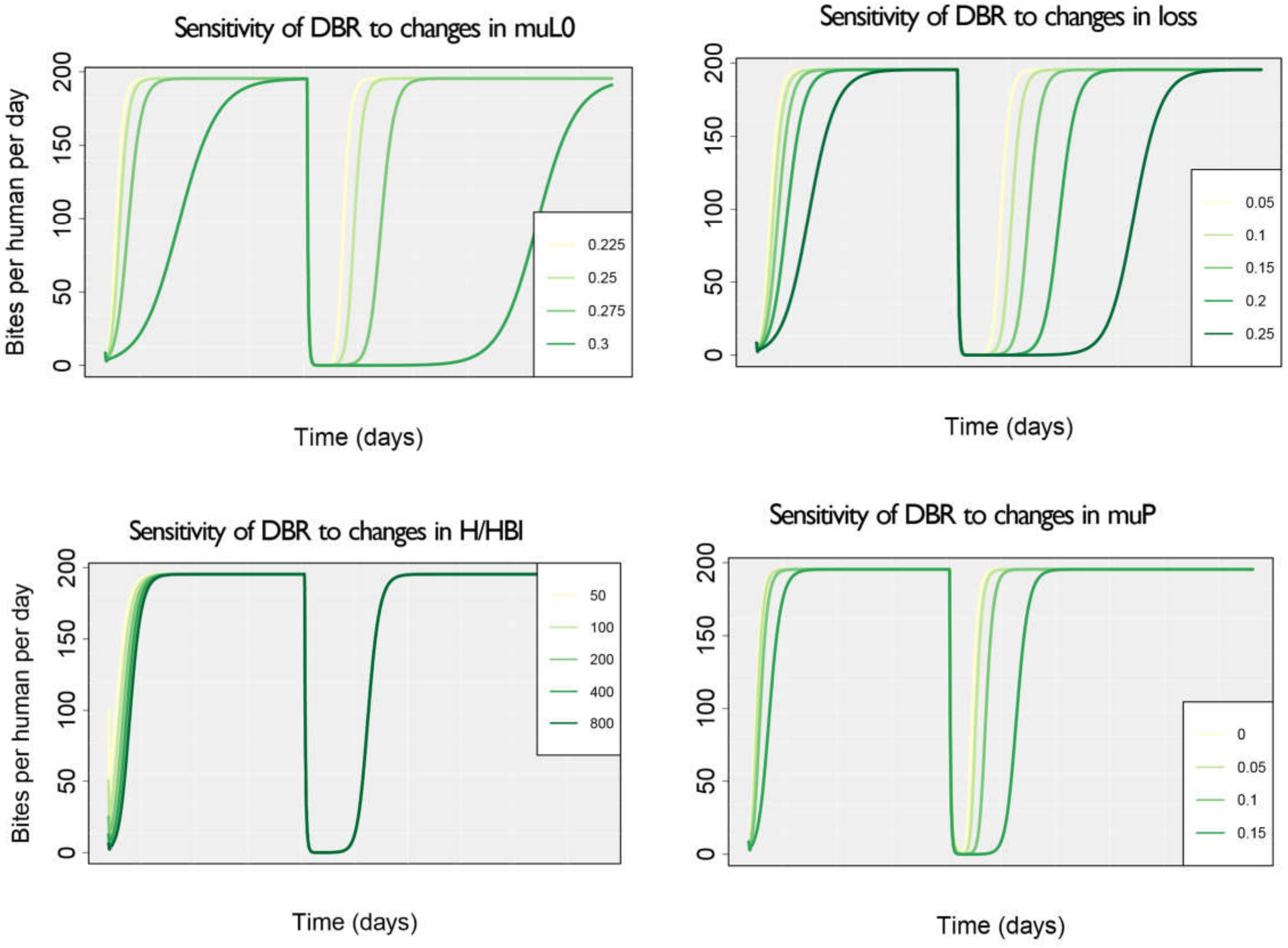

Figure S1 Sensitivity of model output to other parameters not included in Fig 4 of the main text.

4. Pairs plots of parameter estimates

We calculated pairs plots for the 8 estimated parameter values to identify any significant correlations (Figure S2). For *S. squamosum* B/*S. yahense* forest contexts, a negative correlation between the adult loss function and baseline larval mortality was observed. This is because no relationship between temperature and adult mortality is known for this context, so baseline larval mortality and adult mortality are not fully identifiable.

a *S. damnosum* s.s./*S. sirbanum* (savannah)

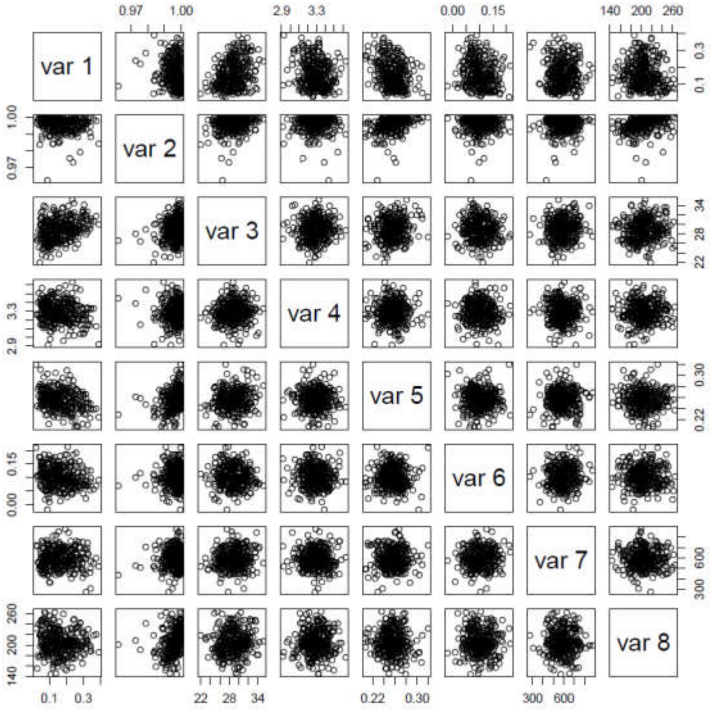

b *S. squamosum* B/*S. yahense* (forest)

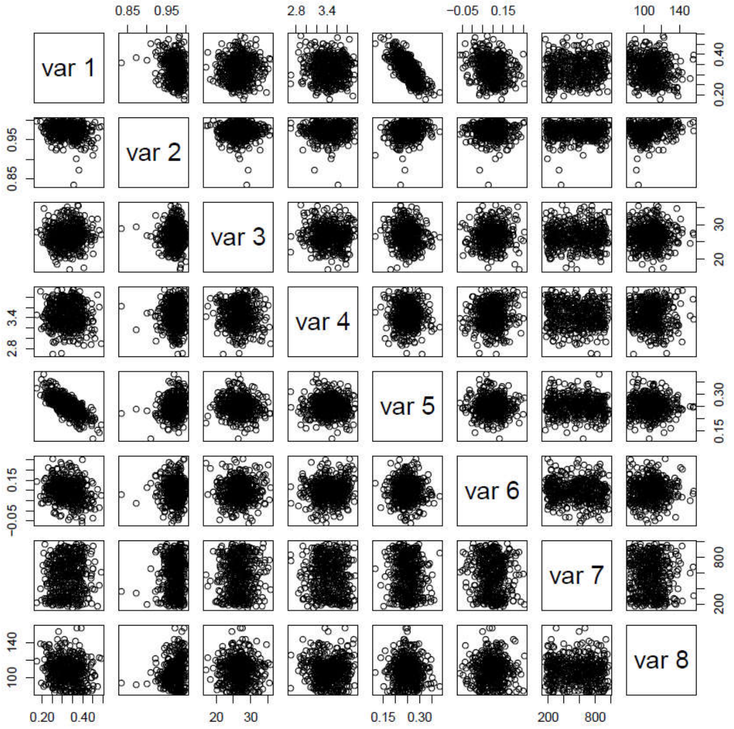

Figure S2 Pairs plots of estimated parameter values for **a)** *S. damnosum s.s./S. sirbanum* (savannah) and **b)** *S. squamosum* B/*S. yahense* (forest). Parameters are denoted as

follows: var 1, per capita loss rate of adult female flies, $\overline{\mu}_V$; var 2, larvicidal efficacy, ε_L; var 3, air temperature, T; var 4, length of gonotrophic cycle, g; var 5, pre-intervention (baseline) larval mortality rate, μ_L^0; var 6, per capita pupal mortality rate, μ_P; var 7, human population density/human blood index, H/h; var 8, pre-intervention (baseline) daily biting rate, DBR^*.

5. Results of scenario analysis

Table S1 Proportion of bites averted when varying larvicidal efficacy, number of treatments and interval between treatments for the savannah- and forest- parameterised SIMPOP model

**Number of treatments**		**4**		**6**		**8**		**10**	
Context		*sav*[a]	*for*[b]	*sav*[a]	*for*[b]	*sav*[a]	*for*[b]	*sav*[a]	*for*[b]
Efficacy (%)	Interval (days)								
99	7	0.4489	NA	0.5654	NA	0.6406	NA	0.6939	NA
	14	0.3879	NA	0.4807	NA	0.5464	NA	0.5952	NA
	21	0.3977	NA	0.4943	NA	0.5609	NA	0.6110	NA
96	7	NA	0.4184	NA	0.4985	NA	0.5568	NA	0.6015
	14	NA	0.3821	NA	0.4517	NA	0.5019	NA	0.5428
	21	NA	0.3766	NA	0.4416	NA	0.4879	NA	0.5223
93	7	0.3431	0.3740	0.4144	0.4500	0.4687	0.5057	0.5119	0.5500
	14	0.3178	0.3454	0.3812	0.4103	0.4283	0.4574	0.4656	0.4961
	21	0.3099	0.3353	0.3659	0.3939	0.4056	0.4358	0.4356	0.4658
70	7	0.2051	0.2147	0.2547	0.2628	0.2944	0.3020	0.3270	0.3342
	14	0.1888	0.1949	0.2277	0.2333	0.2572	0.2618	0.2797	0.2835
	21	0.1757	0.1806	0.2056	0.2097	0.2260	0.2288	0.2403	0.2434

[a] savannah; [b] forest.

Table S2 Proportional reduction in DBR when varying larvicidal efficacy, number of treatments and interval between treatments for the savannah- and forest- parameterised SIMPOP model

**Number of treatments**		**4**		**6**		**8**		**10**	
Context		*sav*[a]	*for*[b]	*sav*[a]	*for*[b]	*sav*[a]	*for*[b]	*sav*[a]	*for*[b]
Efficacy (%)	Interval (days)								
99	7	0.9701	NA	0.9919	NA	0.9977	NA	0.9993	NA
	14	0.7919	NA	0.8489	NA	0.8781	NA	0.8953	NA
	21	0.7165	NA	0.75803	NA	0.7761	NA	0.7847	NA
96	7	NA	0.8921	NA	0.9506	NA	0.9758	NA	0.9877
	14	NA	0.706	NA	0.7779	NA	0.8172	NA	0.8413
	21	NA	0.6227	NA	0.6734	NA	0.6973	NA	0.7095
93	7	0.8382	0.82582	0.9068	0.9026	0.941	0.9412	0.9604	0.9629
	14	0.6217	0.6331	0.6789	0.7042	0.7068	0.7427	0.7216	0.7659
	21	0.5092	0.5392	0.5385	0.5837	0.5489	0.6033	0.5526	0.6126
70	7	0.5091	0.4612	0.5923	0.5527	0.6421	0.6093	0.6739	0.6477
	14	0.3357	0.3197	0.3719	0.3692	0.3879	0.38627	0.3951	0.398
	21	0.2466	0.2434	0.2601	0.2632	0.264	0.2703	0.2652	0.273

[a] savannah; [b] forest.

Table S3 Time to bounce-back (days) of adult fly population when varying larvicidal efficacy, number of treatments and interval between treatments for the savannah- and forest-parameterised SIMPOP model

**Number of treatments**		**4**		**6**		**8**		**10**	
Context		*sav*[a]	*for*[b]	*sav*[a]	*for*[b]	*sav*[a]	*for*[b]	*sav*[a]	*for*[b]
Efficacy (%)	Interval (days)								
99	7	175	NA	199	NA	223	NA	244	NA
	14	142	NA	150	NA	154	NA	158	NA
	21	130	NA	135	NA	137	NA	138	NA
96	7	NA	160	NA	181	NA	199	NA	190
	14	NA	134	NA	144	NA	151	NA	142
	21	NA	121	NA	127	NA	130	NA	120
93	7	143	147	156	164	165	178	173	190
	14	122	124	128	133	131	139	132	142
	21	109	111	112	116	113	118	113	120
70	7	108	99	116	111	121	118	125	123
	14	91	83	96	89	97	92	98	94
	21	78	69	80	72	81	74	81	74

[a] savannah; [b] forest.

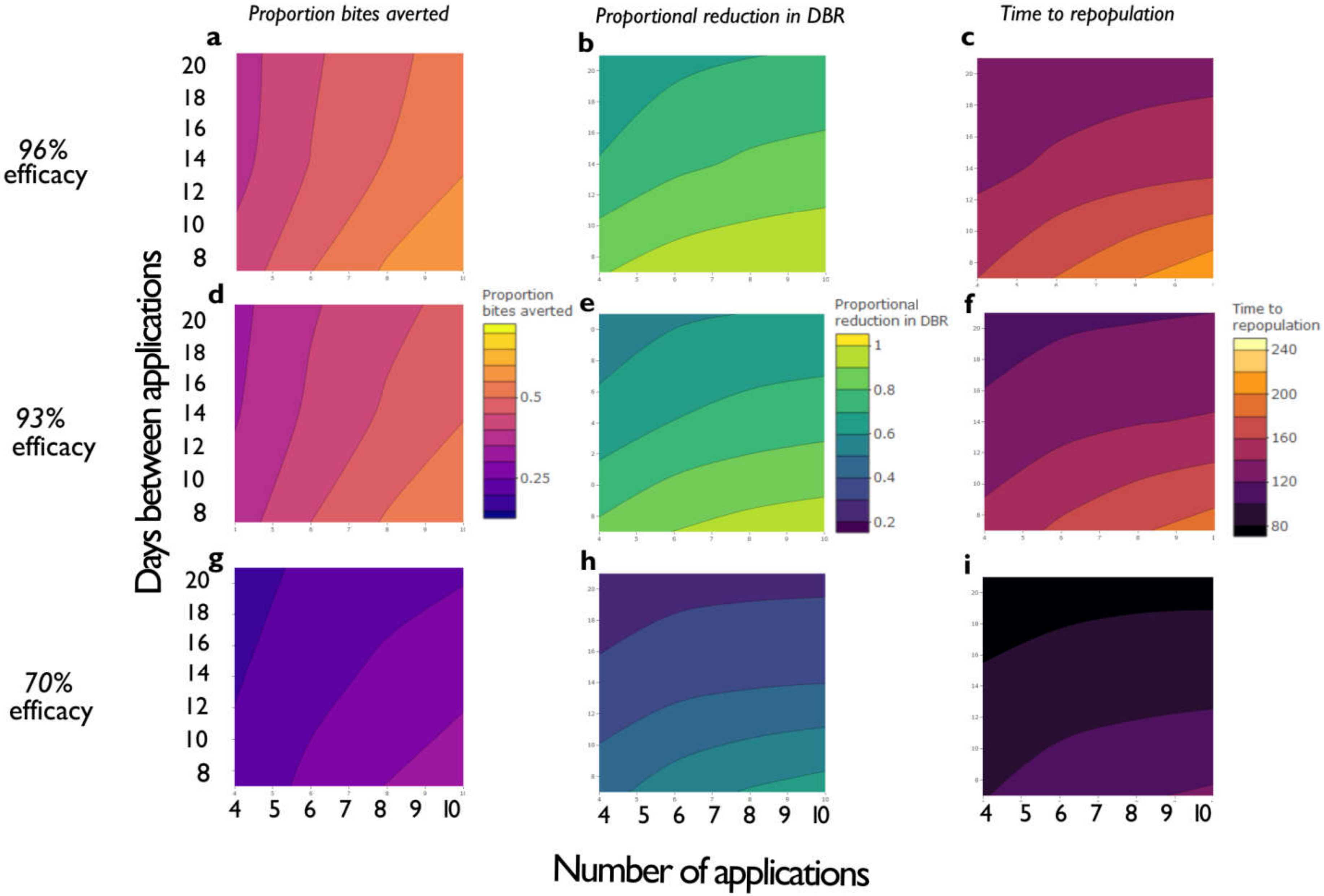

Proportion bites averted
Proportional reduction in DBR
Time to repopulation
a
b
c
d
e
f
g
h
i
96% efficacy
93% efficacy
70% efficacy
Days between applications
Number of applications
Proportion bites averted
0.5
0.25
Proportional reduction in DBR
1
0.8
0.6
0.4
0.2
Time to repopulation
240
200
160
120
80

Figure S3 Scenario analysis (forest settings)

Impact of varying the number of larvicide applications (horizontal axes) and the interval between applications (in days, vertical axes) on three measures of effectiveness: 1) the proportion of bites averted during the intervention (vertical left-hand panels); 2) the proportional reduction in *DBR* (vertical middle panels); 3) the time taken to return to pre-intervention baseline *DBRs* (vertical right-hand panels). In **a)** and **b)** and **c)** larvicidal efficacy is 96%. In **d)**, **e)** and **f)** the results for 93% efficacy are presented. In **g)**, **h)**, and **i)** larvicidal efficacy is 70%. (For precise definitions of the effectiveness measurements see section on Scenario analysis in the main text. Equivalent results for the savannah settings are presented in Fig 3 of the main text.

6. Supplementary references

1. Cheke RA, Basáñez MG, Perry M, White MT, Garms R, Obuobie E, et al. Potential effects of warmer worms and vectors on onchocerciasis transmission in West Africa. Philos. Trans. R. Soc. Lond. B. Biol. Sci. 2015;370(1665):20130559.

2. Csilléry K, François O, Blum MGB. Abc: An R package for approximate Bayesian computation (ABC). Methods Ecol. Evol. 2012;3(3):475–79.

3. Blum MGB, François O. Non-linear regression models for Approximate Bayesian Computation. Stat Comput. 2010;20(1):63–73.

4. Venables WN, Ripley BD. Modern Applied Statistics with S-PLUS. Springer Science & Business Media; 2013.

5. Beaumont MA, Zhang W, Balding DJ. Approximate Bayesian computation in population genetics. Genetics. 2002;162(4):2025–35.
